# Design, Expression, and Binding Interactions Study of the Recombinant Engineered IL-6R

**DOI:** 10.5812/ijpr-165026

**Published:** 2025-11-16

**Authors:** Elham Mehdizadeh Marzenaki, Mojgan Bandehpour, Adel Haghighi, Sepideh Ghani, Maryam Tabarzad, Mahya Nasrollahi, Bahram Kazemi

**Affiliations:** 1 Department of Molecular Medicine, School of Advanced Technologies, Shahid Beheshti University of Medical Sciences, Tehran, Iran; 2 Department of Medical Biotechnology, School of Advanced Technologies in Medicine, Shahid Beheshti University of Medical Sciences, Tehran, Iran; 3 Cellular and Molecular Biology Research Center, Shahid Beheshti University of Medical Sciences, Tehran, Iran; 4 Department of Pathology, Faculty of Specialized Veterinary Sciences, Islamic Azad University, Sciences and Research Branch, Tehran, Iran; 5 Protein Technology Research Center, Shahid Beheshti University of Medical Sciences, Tehran, Iran

**Keywords:** Molecular Docking, IL-6, Autoimmune Diseases, Recombinant Protein Expression

## Abstract

**Background:**

The trans-signaling pathway mediated by soluble interleukin-6 receptor (IL-6R) plays a crucial role in the pathogenesis of chronic inflammatory diseases, autoimmune disorders, and various cancers.

**Objectives:**

The present study aimed to model amino acid residues 121 to 300 of the IL-6R and to predict the effect of this selected fragment on reducing its interaction with glycoprotein 130 (gp130) using molecular docking.

**Methods:**

The engineered selected sequence interleukin-6 receptor (seIL-6R) was designed to diminish interaction with gp130. Physicochemical parameters were evaluated using the ProtParam tool. Structural modeling and prediction were performed using AlphaFold. Molecular docking was conducted using ClusPro. Subsequently, the seIL-6R gene was recombinantly expressed in the Chinese hamster ovary (CHO)-K1 cell line. The expression of recombinant seIL-6R was evaluated by Western blotting, and its secondary structure was examined by Fourier transform infrared (FTIR) spectroscopy.

**Results:**

Evaluation of the physicochemical parameters of the recombinant seIL-6R protein demonstrated improved stability and solubility, along with a reduced molecular weight (20.6 kDa). Molecular docking results indicated reduced binding of seIL-6R to gp130. Expression of the recombinant seIL-6R protein was confirmed by Western blotting. Furthermore, FTIR spectroscopy revealed that the secondary structure of seIL-6R was preserved and consistent with predicted structural models.

**Conclusions:**

This engineered protein has potential for further investigation as a promising and cost-effective therapeutic agent targeting IL-6-related pathologies.

## 1. Background

The cytokine interleukin-6 (IL-6) is a central mediator in autoimmune and inflammatory diseases. Understanding the molecular interactions involving IL-6, particularly at the amino acid level, is critical for the development of IL-6-inhibiting therapeutic compounds (1). Characterization of the interleukin-6 receptor (IL-6R) complex has clarified its structural and signaling roles, providing essential insights for pharmacotherapeutic innovation. The IL-6 interacts with its specific receptor, IL-6R, and the signal transducer glycoprotein 130 (gp130), which is almost ubiquitously expressed on cell surfaces. The mature IL-6R (80 kDa) is derived from a glycosylated precursor (50 kDa). Motif analysis reveals that the extracellular region of IL-6R comprises three domains: D1, D2, and D3. The N-terminal D1 domain is a hallmark of the immunoglobulin superfamily, while D2 and D3 are homologous to cytokine-binding domains (CBD) containing two fibronectin type III (FnIII) regions (2). Although the D1 domain is not essential for ligand recognition and signal initiation, it contributes to protein stability (3).

The IL-6R exists in two primary forms: The membrane-bound form (mIL-6R) and the soluble form (sIL-6R). The soluble isoforms are generated either through proteolytic cleavage of the membrane-bound receptor or via alternative splicing. These isoforms can bind IL-6 and activate trans-signaling by interacting with the gp130 receptor on cells lacking mIL-6R, thereby activating downstream pathways such as the JAK/STAT cascade, which are crucial for regulating inflammatory and immune responses. Comparative studies of IL-6R isoforms reveal that the soluble isoform promotes broader trans-signaling, while the membrane-bound isoform mediates classic signaling. Clinically, the trans-signaling pathway mediated by sIL-6R plays a significant role in the pathogenesis of chronic inflammatory diseases, autoimmune disorders, and cancers. Its smaller size (approximately 40 kDa) leads to enhanced pharmacokinetic properties, making it a valuable target for therapeutic development (4).

Consequently, targeting IL-6 trans-signaling has emerged as a novel therapeutic strategy. For example, monoclonal antibodies that specifically inhibit sIL-6R or the IL-6/sIL-6R complex have demonstrated efficacy in reducing inflammation and disease progression (5). Biologics targeting inflammatory cytokines or their associated signaling pathways are widely used to treat chronic inflammatory diseases (6). Unlike small-molecule immunosuppressants and steroids, which broadly suppress immune activity, biologics can achieve clinical remission. Tocilizumab, a humanized monoclonal antibody that inhibits IL-6R signaling, exemplifies this targeted approach in rheumatoid arthritis (RA) treatment (7). Emerging biologics that disrupt IL-6R signaling in distinct ways are under clinical evaluation, highlighting the varied pathological outcomes of targeting different aspects of the IL-6 signaling axis (1).

## 2. Objectives

In this study, we hypothesized that truncation of IL-6R, while retaining IL-6 binding, may reduce gp130 binding; therefore, amino acid residues 121 to 300 of the IL-6R were selected. The truncated structure of IL-6R was modeled, and its interactions with gp130 were predicted using molecular docking. Additionally, recombinant selected sequence interleukin-6 receptor (seIL-6R) protein was expressed, and its secondary structure was evaluated using Fourier transform infrared (FTIR) spectroscopy.

## 3. Methods

### 3.1. Retrieval of Interlukin-6, Glycoprotein 130 Structures, and Interleukin-6 Receptor/Interleukin-6 Receptor Fragment Sequences

The three-dimensional structures of IL-6, IL-6R, and gp130 were obtained from the Protein Data Bank. The sequence of IL-6Rα, comprising 180 residues (from 121 to 300), was retrieved from the UniProt database for analysis (8).

### 3.2. Evaluation of Physicochemical Parameters and Solubility

Physicochemical properties, including molecular weight, half-life, Instability Index, Aliphatic Index, and the grand average of hydropathicity (GRAVY), were evaluated for both the intact and selected seIL-6R using the ProtParam tool (9). Protein solubility was assessed using the Protein-Sol server (10).

### 3.3. Prediction of Secondary and Tertiary Structures

Secondary structure predictions for seIL-6R and IL-6R were performed using the secondary structure prediction method (SOPMA) server, which predicts four conformational states: Helices, beta-sheets, turns, and random coils (Ccs). Three-dimensional structures were predicted using the AlphaFold server (11) and used as templates for further analysis.

### 3.4. Evaluation and Refinement of Structural Models

The quality and validity of the AlphaFold-generated models for seIL-6R and IL-6R were evaluated using Ramachandran plots (12) and ProSA-web plots (13). Structural refinement was performed using the GalaxyRefine server (14).

### 3.5. Molecular Docking Analysis Among Interlukin-6, Selected Sequence Interleukin-6 Receptor, Interleukin-6 Receptor, and Glycoprotein 130

Molecular docking was conducted using the ClusPro 2.0 web server (15) to identify key residues involved in the interactions between gp130, IL-6, and IL-6Rα in both the selected sequence and native forms. Initially, docking of IL-6 with IL-6Rα was performed using default parameters, and the model with the lowest energy was selected. The resulting complex was subsequently docked with gp130. Protein Data Bank (PDBsum) (16) was used to identify interacting residues, and all 3D visualizations were performed using Chimera software (17).

### 3.6. Construction Design and Codon Optimization of Selected Sequence Interleukin-6 Receptor Sequence

**Figure 1.**
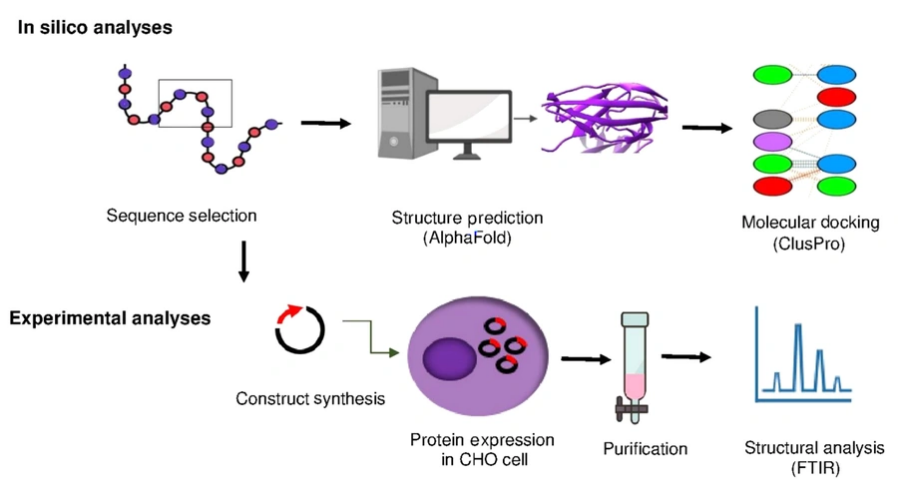
Schematic figure of design, computational analysis and recombinant expression of truncated interleukin-6 receptor (seIL-6R).

The engineered amino acid sequence of seIL-6R was reverse-translated into a DNA sequence. The sequence was optimized for cloning into a eukaryotic pIRES2-EGFP vector (in the Supplementary File 1). Restriction sites were identified using the GeneRunner and SnapGene 3.2.1 tools. The optimized sequence, including restriction sites, Kozak sequence, an upstream signal peptide from the human serum albumin gene, and a downstream S-tag peptide, stop codon, and poly(A) signal, was inserted into the pIRES2-EGFP plasmid between SacI and SalI restriction sites under the CMV promoter (in the Supplementary File 2) (Figure 1). 

### 3.7. Expression of Selected Sequence Interleukin-6 Receptor Recombinant Protein

The seIL-6R gene was cloned into the pIRES2-EGFP vector by GeneRay Biotechnology Co. (China). The plasmid, pIRES2-EGFP-seIL-6R, was propagated in *Escherichia coli* Top10 and extracted using the QIAGEN Plasmid Mini Kit (Cat. No. 27106). Plasmid accuracy was confirmed by restriction enzyme analysis using BglII (Fermentas, Lithuania). Chinese hamster ovary (CHO)-K1 cells (ATCC^®^ CCL-61), adapted for suspension culture, were maintained in serum-free medium (Gibco CHO-S-SFMII, Cat. No. 11580416) and incubated in a humidified 5% CO_2_ incubator at 37°C for 24 hours. Cells were transfected by electroporation (400 V, 200 µs, 2 pulses) with 20 µL (617 ng/µL) of the pIRES2-EGFP-seIL-6R plasmid. After recovery in serum-free medium, green-fluorescent protein (GFP) expression was observed using fluorescence microscopy (Olympus Model CKX41SF). G418 (100 µg/mL) was added to the culture medium to select transfected cells. Cells surviving antibiotic selection, indicating successful integration or maintenance of the plasmid, were considered transfectants (Figure 1). 

### 3.8. Recombinant Protein Purification and Confirmation by Western Blotting

- Protein purification: Recombinant seIL-6R protein was purified using an S-protein agarose column (Millipore, USA) according to the manufacturer’s protocol (18). The purified protein was dialyzed against PBS (pH 7.4) at 4°C for 8 hours, concentrated using Amicon ultra-centrifugal filters (Merck, Germany), and quantified using the Bradford assay (19). Purity was confirmed by sodium dodecyl sulfate-polyacrylamide gel electrophoresis (SDS-PAGE) (12% gel) and Coomassie blue staining.

- Western blotting: Protein bands were transferred onto a nitrocellulose membrane, blocked with 3% skim milk (Fluka, Switzerland), and incubated with an alkaline phosphatase-conjugated anti-S-tag antibody (Abcam, UK; 1:2000 dilution) at room temperature for 2 hours. Protein bands were visualized using nitro blue tetrazolium/5-bromo-4-chloro-3-indolyl-phosphate (NBT/BCIP) substrate solution (Roche, Germany).

### 3.9. Fourier Transform Infrared Spectroscopy Analysis

The FTIR spectroscopy was used to analyze the secondary structure of recombinant seIL-6R protein. Samples were prepared by lyophilizing the purified protein and mixing approximately 1 mg of the dry sample with 100 mg of spectroscopic-grade potassium bromide (KBr). The FTIR spectra were recorded using a PerkinElmer Spectrum One spectrometer (Explorer-GNR Company, Italy) in the range of 4000 - 400 cm^-1^ at room temperature, with a resolution of 4 cm^-1^ and 32 scans per sample. The resulting spectra were analyzed to identify characteristic absorption bands corresponding to functional groups and secondary structural elements of the protein, such as amide I and amide II bands.

## 4. Results

### 4.1. Design of Selected Sequence Interleukin-6 Receptor

The three-dimensional structures of IL-6 (PDB ID: 1ALU) and gp130 (PDB ID: 3L5H) were retrieved from the Protein Data Bank. Isoform two of IL-6R (UniProtKB: P08887) was selected as the reference sequence. The selected sequence, consisting of 180 residues (from 121 to 300), was registered in the GenBank database with accession number PX289565.

### 4.2. Evaluation of Selected Sequence Interleukin-6 Receptor Physicochemical Parameters and Solubility

Table 1 presents the physicochemical parameters for the intact extracellular domain and the selected sequence of IL-6Rα. The molecular weight was reduced in the selected sequence compared to the intact form. The isoelectric point increased, while the half-life remained unchanged. The Instability Index was reduced in the smaller form, indicating increased stability. The more negative GRAVY value for the truncated form suggests increased hydrophilicity. Solubility analysis revealed values of 0.434 and 0.286 for the selected sequence and intact forms, respectively.

**Table 1.** Physicochemical Properties of Intact and Selected Forms of Interleukin-6 Receptor as Assessed by the ProtParam Tool

Physicochemical Parameters	Extracellular Domain of IL-6R	seIL-6R
**Molecular weight **	40237.50	20741.53
**Numbers of amino acid**	365	180
**Theoretical pI**	8.25	8.90
**Formula**	C1767H2752N514O529S18	C923H1410N260O267S10
**Total numbers of the residues with positive charge**	37	20
**Total numbers of residues with negative charge**	34	15
**Expected half-life**	(*Escherichia coli*, in-vivo) > 10 h	(*E. coli*, in-vivo) > 10 h
**Expected half-life**	(yeast in-vivo) > 20	(yeast in-vivo) > 20 h
**Half-life**	Mammalian reticulocytes, in-vitro = 30	Mammalian reticulocytes, in-vitro = 30 h
**Instability Index ** ^**a**^	63.12	56.42
**Aliphatic Index ** ^**b**^	70.82	64.03
**GRAVY ** ^**c**^	-0.370	-0.426

Abbreviations: IL-6R, interleukin-6 receptor; seIL-6R, selected sequence interleukin-6 receptor; pI, isoelectric point; GRAVY, grand average of hydropathicity.

^a^ > 40 unstable; < 40 stable protein.

^b^ Higher values indicate greater thermal stability.

^c^ Values < 0 indicate hydrophilicity; > 0 hydrophobicity.

Secondary structure predictions for seIL-6R and IL-6R were performed using SOPMA software (8). The extracellular domain of IL-6R exhibited 5.75% alpha-helix (Hh), 24.11% extended strand (Ee), and 70.14% random coil (Cs). In contrast, the selected sequence seIL-6R displayed 1.66% Hh, 29.53% Ee, and 68.51% Cs. This indicates a decrease in Cs and Hh content and an increase in Ee for the selected sequence form (in the Supplementary File 3).

**Figure 2.**
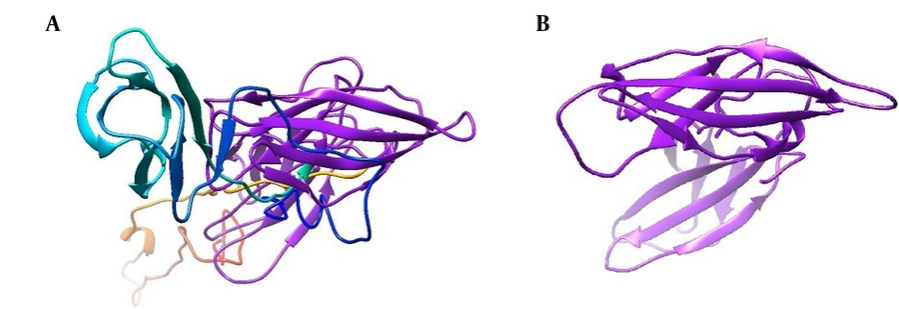
Tertiary structures predicted by AlphaFold 2.0. A, extracellular domain of interleukin-6 receptor (IL-6R) (pLDDT = 81, pTM = 0.707); B, selected sequence as selected sequence interleukin-6 receptor (seIL-6R) (pLDDT=93.2, pTM=0.814). Models with pLDDT > 90 and pTM > 0.5 are considered reliable.

The three-dimensional structure of the selected sequence was predicted using the AlphaFold online server (11) (Figure 2). The model is available in ModelArchive at http://www.modelarchive.org/doi/10.5452/ma-vhird. Evaluation metrics include pLDDT, an Accuracy Index (values > 70 indicate high confidence), and pTM, a similarity measure (values close to 1 indicate high structural similarity).

### 4.3. Evaluation and Refinement of Models

**Figure 3.**
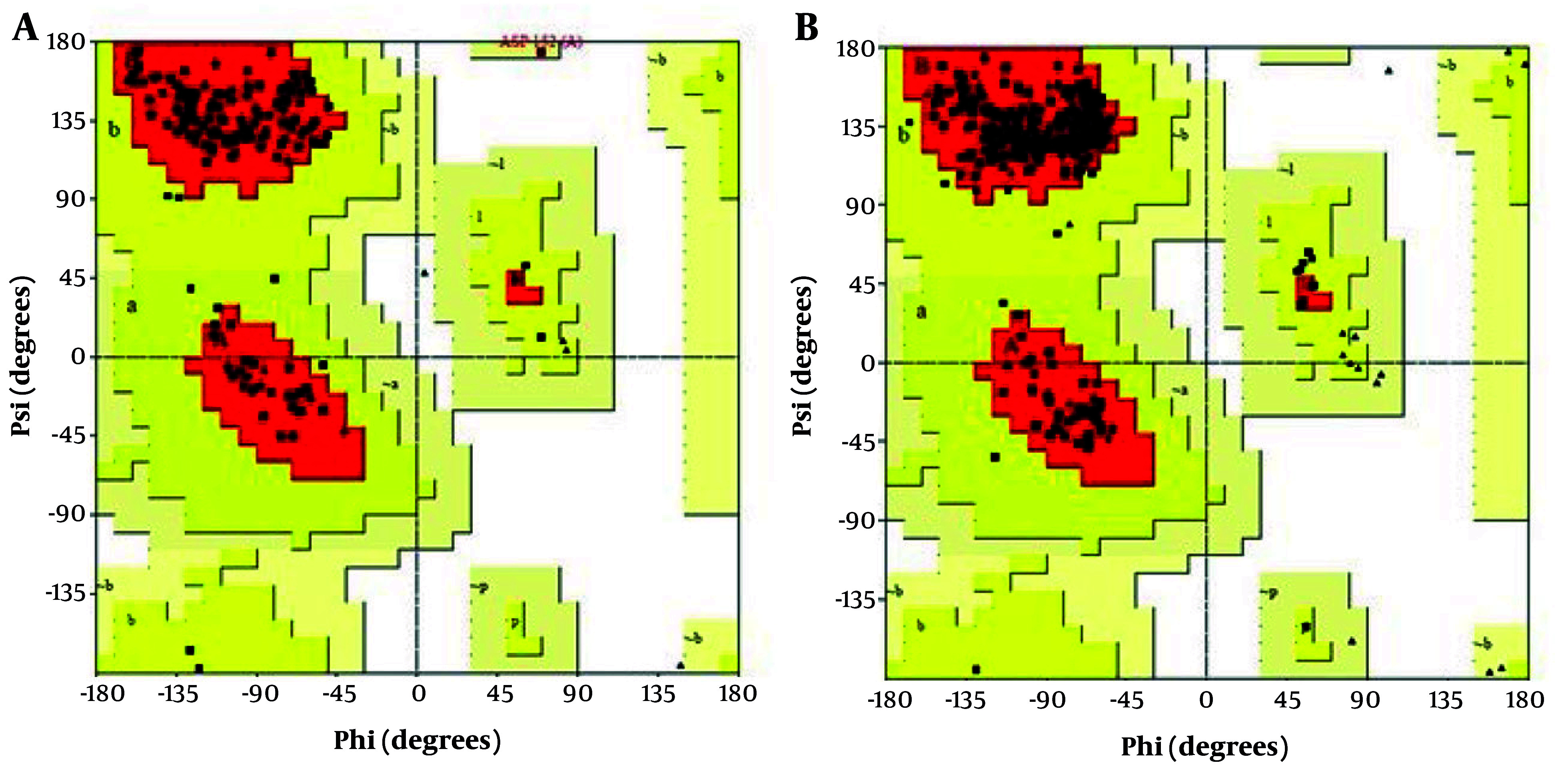
Ramachandran plot validation using the PROCHECK server. A, extracellular domain of interleukin-6 receptor (IL-6R) after refinement; B, selected sequence, selected sequence interleukin-6 receptor (seIL-6R). The percentage of residues in favorable regions is 95.2% for seIL-6R compared to 74.3% for the extracellular domain of IL-6R.

Ramachandran plot analysis revealed that 92.5% of the residues in the selected seIL-6R model were located in favorable regions, requiring no further refinement (Figure 3). For the intact IL-6R model, only 74.3% of residues were in favorable regions; after refinement, this increased to 95.2% for seIL-6R.

ProSA server analysis provided z-scores indicating structural quality. The intact IL-6R had a z-score of -5.31, and the selected sequence seIL-6R had a z-score of -5.41. Both scores are within the expected range for native proteins of similar size (in the Supplementary File 4).

### 4.4. Molecular Docking of Interleukin-6 Receptor/Interleukin-6 and Selected Sequence Interleukin-6 Receptor/Interleukin-6 with Glycoprotein 130

**Figure 4.**
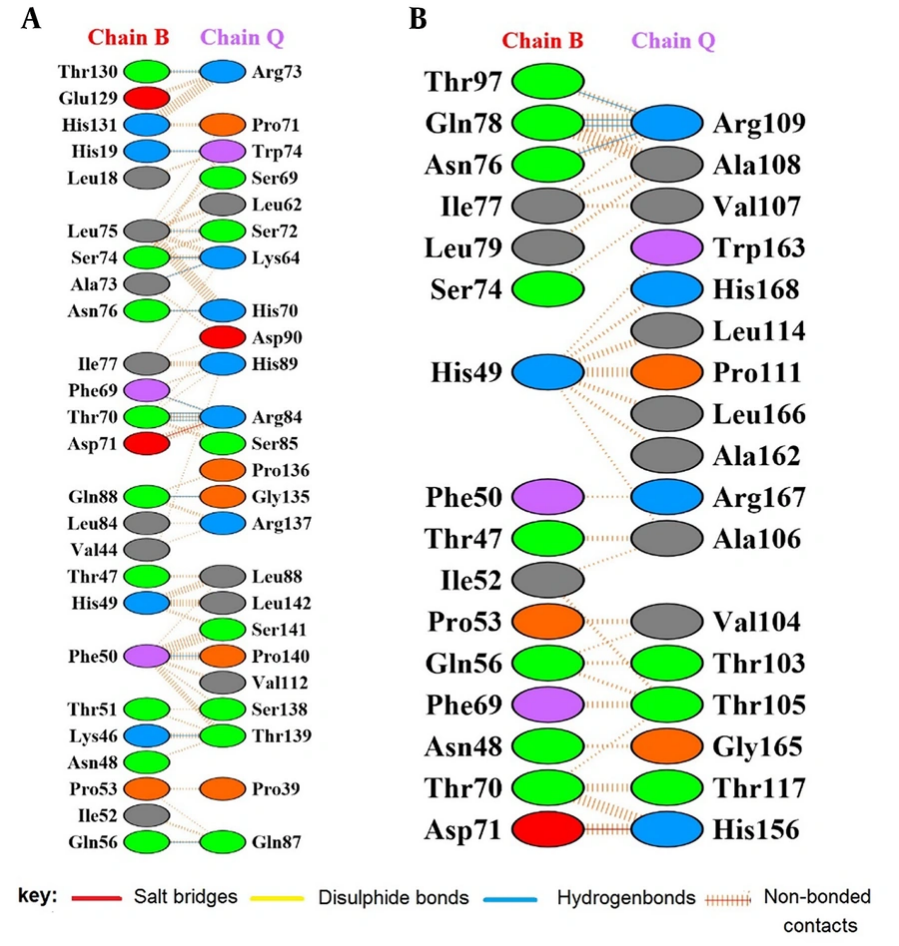
Protein interactions extracted by Protein Data Bank (PDBsum) after protein-protein docking. A, extracellular domain of interleukin-6 receptor (IL-6R) (365 aa, chain Q) and glycoprotein 130 (gp130) (chain B); B, selected sequence selected sequence interleukin-6 receptor (seIL-6R) (180 aa, chain Q) and gp130 (chain B).

Molecular docking was performed using the ClusPro 2.0 server. The best model, with the lowest energy (-844.9), was selected among the 20 models generated by the server. Interacting residues of IL-6R (365 aa, Chain Q) and selected sequence seIL-6R (180 aa, Chain Q) with gp130 (Chain B) were identified in the docked model using the PDBsum program (Figure 4). Unlike IL-6R, which forms 14 hydrogen bonds with gp130, the engineered seIL-6R exhibited a reduction to 4 hydrogen bonds.

### 4.5. Reverse Translation and Codon Optimization of the Selected Sequence Interleukin-6 Receptor Sequence

A 738 bp gene encoding seIL-6R was synthesized and cloned into the pIRES2-EGFP expression vector under the control of the SV40 promoter. The construct was confirmed by double digestion with BglII, resulting in the separation of the coding gene observed at approximately 730 bp (in the Supplementary File 5).

### 4.6. Cloning and Expression of Recombinant Selected Sequence Interleukin-6 Receptor Protein

**Figure 5.**
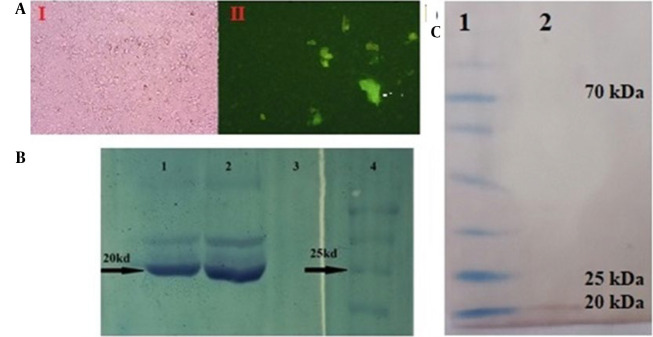
Expression analysis of selected sequence interleukin-6 receptor (seIL-6R) protein. A, green-fluorescent protein (GFP) expression in Chinese hamster ovary (CHO)-K1 cells and secretion in culture medium (Marked with an arrow). (1) light microscopy; (2) immunofluorescence microscopy; B, sodium dodecyl sulfate-polyacrylamide gel electrophoresis (SDS-PAGE) showing a 20 kDa band in lanes 1 and 2. Lane 3, negative control, lane 4, protein marker; C, western blot validation by anti-stag antibody. Lane 1, prestained protein marker; Lane 2, seIL-6R band.

The pIRES2-EGFP-seIL-6R construct was transfected into CHO-K1 cells. The GFP expression confirmed successful transfection, with transient expression observed as protein aggregates in the culture medium within 48 hours (Figure 5A). Stable transfection was achieved in CHO cells under G418 selection.

### 4.7. Recombinant Protein Purification and Western Blot Confirmation

The recombinant seIL-6R protein (~ 20 kDa) was purified under native conditions using S-protein resin and confirmed by SDS-PAGE (Figure 5B) and Western blotting (Figure 5C). 

### 4.8. Fourier Transform Infrared Analysis of Recombinant Selected Sequence Interleukin-6 Receptor Protein

**Figure 6.**
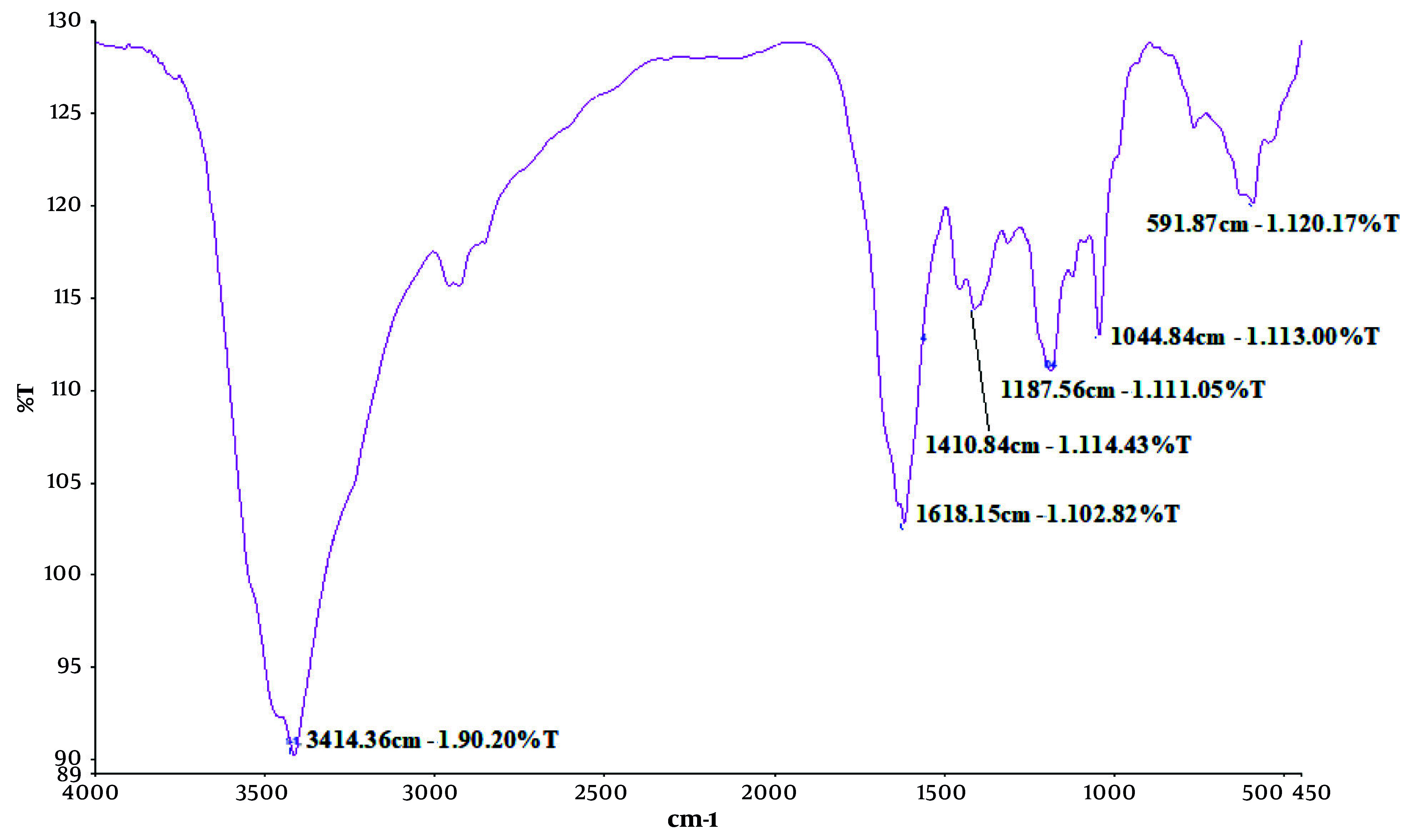
Secondary structure analysis of soluble IL6R (residues 121 - 300) by Fourier transform infrared spectroscopy (FTIR) spectroscopy

The FTIR spectrum was obtained to assess retention of the secondary structure of seIL-6R due to selection of the 121 - 300 sequence of IL-6R (Figure 6). The FTIR spectrum of the recombinant engineered IL-6R protein revealed several distinct absorption peaks corresponding to specific secondary structural elements: A peak at 1618.15 cm^-1^ within the amide I region (1600 - 1700 cm^-1^), primarily due to C=O stretching vibrations in the peptide backbone, suggests the presence of β-sheet structures, typically observed in the range of 1620 - 1640 cm^-1^. The intensity (102.82% T) indicates a moderate contribution of β-sheets to the overall protein structure. Another peak at 1410.84 cm^-1^, slightly shifted from the typical amide II range (1500 - 1600 cm^-1^), may indicate contributions from Cs or unordered structures. Peaks observed at 1187.56 cm^-1^ and 1044.84 cm^-1^ fall within or near the amide III region (1200 - 1400 cm^-1^), associated with C–N stretching and N–H bending, reflecting a mixture of α-helical and β-sheet elements, though these are less definitive than the amide I band. No prominent absorption was observed in the 1650 - 1660 cm^-1^ range, typical for α-helical content, suggesting low α-helix presence.

A broad absorption band at 3414.36 cm^-1^ corresponds to O–H and N–H stretching vibrations, indicating hydrogen bonding, likely from water molecules or polar amino acid side chains. A broader band in the 3310 - 3270 cm^-1^ range was associated with the amide A band, reflecting N–H stretching sensitive to backbone conformation and hydrogen bonding. A weak secondary absorption in the 3100 - 3030 cm^-1^ range may indicate the presence of a Fermi resonance component of amide A. The detailed characteristics of the principal FTIR peaks and the assignment of amide bands are presented in the Supplementary Files 6 and 7.

## 5. Discussion

In this study, we analyzed the role of residues 121 - 300 in the interaction of recombinant seIL-6R with gp130 using molecular docking. This sequence length reduction strategy aimed to reduce molecular weight and complexity while preserving functional domains critical to IL-6 interaction. The resulting protein exhibited reduced molecular weight (20.6 kDa). Molecular docking confirmed that recombinant seIL-6R forms fewer hydrogen bonds with gp130 compared to intact IL-6R.

The 121 - 300 fragment corresponds to the central portion of the extracellular domain of IL-6R and includes key residues involved in IL-6 binding. In protein engineering, deletion of transmembrane and intracellular regions is a common strategy to generate soluble receptor fragments that can be efficiently expressed in heterologous systems such as bacteria, yeast, or mammalian cells. Crystallographic data indicate that the N-terminal immunoglobulin-like domains mainly support ligand interaction but are not strictly required for direct IL-6 binding. Their deletion reduces protein size and improves stability and solubility without compromising functionality (2).

Recent structural and computational studies have clarified the binding mechanisms of IL-6R-targeting antibodies such as tocilizumab and sarilumab, which inhibit IL-6 signaling by competitive binding at the D3 domain (20). Docking analyses have confirmed the higher affinity of such antibodies compared to native ligands (21). Despite clinical success, monoclonal antibody therapies present challenges, including high costs and adverse effects, motivating the development of small molecule inhibitors and engineered receptor fragments as potential alternatives (22). Therefore, the selected fragment (121 - 300) and deletion of residues 0 - 120 and 301 - 360 could achieve the optimal balance between minimal size and preserved activity, and align with these trends by providing a truncated fragment. Moreover, our study builds on previous research identifying amino acids critical for IL-6R binding to IL-6 and gp130; essential residues such as L247, F248, R250, Q255, and H256 were preserved in the engineered protein sequence to maintain binding interaction (23).

Studies have shown that IL-6 can have anti-inflammatory or pro-inflammatory effects depending on its signaling pathway. Classical IL-6 signaling (via the membrane receptor IL-6R) has predominantly anti-inflammatory effects. However, trans-signaling (via the soluble receptor sIL-6R, which binds to IL-6 and signals with gp130 on other cells) induces pro-inflammatory effects and inflammation. Soluble gp130 acts as an antagonist of this trans pathway and can inhibit inflammation (24, 25). Therefore, by engineering the soluble IL-6R receptor and reducing its binding to gp130, our study could lead to a reduction in inflammation through a similar mechanism.

The FTIR analysis of the recombinant IL-6R protein revealed key structural features. The peak at 1618.15 cm^-1^ (amide I band) indicates a moderate presence of β-sheet structures, in agreement with SOPMA predictions. Hydrogen bonding was confirmed by the broad 3414.36 cm^-1^ band and the amide A region (3310 - 3270 cm^-1^), suggesting proper folding and backbone stability. These findings confirm that the engineered IL-6R adopts a biologically relevant conformation, with dominant β-sheet and low α-helical content, consistent with its expected binding and functional properties (26).

In this study, we engineered a truncated IL-6 receptor fragment (residues 121 - 300) for the first time. Unlike previous approaches focusing on full-length IL-6R, this targeted construct enables precise investigation of IL-6 binding domains and provides a potential therapeutic antagonist to modulate inflammatory responses. Given the central role of IL-6 in RA and other chronic inflammatory diseases, this strategy offers a promising, cost-effective alternative to current monoclonal antibody therapies (27).

### 5.1. Conclusions

Molecular docking was employed to predict the structure of the truncated IL-6R and its interactions with gp130. Our findings demonstrated that recombinant seIL-6R, compared with intact IL-6R, exhibits reduced hydrogen bonding and other interactions with gp130. Although these results provide valuable insights, further experimental validation through functional assays of recombinant seIL-6R is recommended. This engineered protein has the potential to be further studied as a promising therapeutic agent targeting IL-6-related pathologies.

## supplementary material

ijpr-24-1-165026.pdf

## Data Availability

The data presented in this study are uploaded during submission as a supplementary file and are openly available for readers upon request.
